# Histone modifications in skin fibrosis: linking immune dysregulation, metabolic reprogramming, and persistent fibrotic remodeling

**DOI:** 10.3389/fimmu.2026.1865170

**Published:** 2026-06-18

**Authors:** Yuezhong Chen, Junzhe Chen, Ziyi Luo, Shaoxiang Yuan, Tao Xiong, Yan Zhou, Kechen Ye, Shune Xiao, Chengliang Deng

**Affiliations:** Department of Burns and Plastic Surgery, Affiliated Hospital of Zunyi Medical University, Zunyi, Guizhou, China

**Keywords:** epigenetic reprogramming, fibroblast activation, histone lactylation, histone modifications, immune dysregulation, skin fibrosis

## Abstract

Skin fibrosis encompasses a spectrum of disorders, including hypertrophic scars, keloids, systemic sclerosis and lymphedema, that arise from distinct initiating insults but converge on persistent fibroblast activation, immune dysregulation and excessive extracellular matrix deposition. Although inflammatory, metabolic and mechanical abnormalities are increasingly recognized in these diseases, the mechanisms through which these signals are integrated into sustained profibrotic transcriptional programs remain incompletely understood. Histone modifications provide a dynamic and potentially reversible regulatory layer that links changes in the tissue microenvironment to chromatin accessibility and gene expression. In this Review, we summarize the major classes of histone modifications implicated in skin fibrosis, including acetylation, methylation, lactylation and selected non-canonical modifications. We discuss how their writers, erasers and readers regulate fibroblast activation, immune-cell dysfunction and profibrotic signaling pathways, and compare the available evidence across pathological scars, systemic sclerosis and lymphedema. Current evidence is most extensive for acetylation-related mechanisms, whereas the effects of histone methylation are highly dependent on the modified locus, cell type and experimental context. Histone lactylation provides an emerging mechanistic link between glycolytic reprogramming, lactate accumulation and profibrotic transcription, although its broader importance across cutaneous fibrotic diseases remains to be established. We further evaluate inconsistencies among experimental studies, limitations in causal interpretation and the translational challenges associated with targeting histone-modifying enzymes and chromatin readers. These challenges include limited cell-type specificity, non-histone effects, systemic toxicity and inadequate delivery to fibrotic tissues. Future integration of longitudinal, cell-resolved and spatial epigenomic approaches may help define disease-specific chromatin programs and facilitate the development of more precise biomarkers and targeted antifibrotic therapies.

## 1 Introduction

Skin fibrosis encompasses a heterogeneous group of disorders, including hypertrophic scars (HS), keloids, systemic sclerosis (SSc) and lymphedema. These diseases arise from distinct initiating events, such as aberrant wound healing, autoimmune and vascular injury, or impaired lymphatic drainage, but converge on persistent fibroblast activation, myofibroblast accumulation and excessive extracellular matrix (ECM) deposition (1–3). Inflammation, immune-cell recruitment and profibrotic growth-factor signaling contribute substantially to disease progression. However, these pathways alone do not fully explain how inflammatory, metabolic and mechanical abnormalities are integrated into durable transcriptional programs that sustain fibrotic tissue remodeling.

Epigenetic regulation provides a potential molecular framework for understanding this persistence. Epigenetic mechanisms modulate gene expression without altering the underlying DNA sequence and include DNA methylation, histone modifications, chromatin remodeling and non-coding RNA-mediated regulation (4, 5). Among these mechanisms, histone modifications are particularly relevant because they are dynamically deposited, removed and interpreted by writers, erasers and readers. Through these processes, histone modifications regulate chromatin accessibility, transcription-factor recruitment and gene expression while remaining responsive to changes in cellular signaling and metabolism (6–8).

Histone acetylation and methylation have been extensively investigated in inflammation, immunity and fibrosis. Acetylation generally promotes chromatin accessibility, although its biological effects depend on the modified substrate and the activity of associated chromatin readers. Histone methylation can either activate or repress transcription according to the modified residue and methylation state. More recently, histone lactylation has expanded the conceptual scope of epigenetic regulation by linking lactate metabolism to chromatin-level transcriptional control (9, 10). This mechanism is potentially relevant to fibrotic skin, where hypoxia, persistent inflammation, altered glycolysis and mechanical stress coexist within the pathological microenvironment.

Despite these advances, the available evidence remains fragmented. Most studies have focused on individual enzymes, selected modification sites or single diseases, and findings from patient tissues, cultured cells and animal models are frequently discussed without sufficient distinction between descriptive association and functional evidence. It therefore remains unclear which findings are broadly shared across cutaneous fibrotic diseases, which reflect disease-specific or cell-type-specific programs, and which discrepancies arise from differences in experimental models, disease stages or pharmacological strategies. The relationships among acetylation, methylation, lactylation and other chromatin modifications also remain incompletely defined. Rather than forming a uniform linear pathway, these modifications may act as interacting and context-dependent regulatory layers (11–13).

Histone lactylation illustrates both the potential and the current limitations of this field. Recent studies in pathological scars have identified locus-specific H3K18la- and H3K23la-associated mechanisms linking lactate metabolism to fibroblast activation (14, 15). However, these findings are derived from a limited number of experimental systems and have not yet established lactylation as a universal or dominant mechanism across skin fibrotic diseases. Similarly, most existing studies rely on bulk tissues or isolated cell populations, which limits the ability to distinguish histone modification patterns among fibroblast subsets, immune cells, vascular cells and lymphatic endothelial cells or across spatially distinct fibrotic niches.

In this Review, we summarize and critically evaluate the roles of histone acetylation, methylation, lactylation and selected non-canonical modifications in pathological scars, SSc and lymphedema. We compare consistent and contradictory findings, assess the relative strength of the available evidence and discuss how histone modifications interact with inflammatory, metabolic and mechanical signals. We also examine the limitations of current experimental models and therapeutic strategies and outline how longitudinal, single-cell and spatial epigenomic approaches may advance mechanistic understanding and clinical translation.

## 2 Histone modifications and their functions

### 2.1 Histone acetylation

Histone acetylation is a post-translational modification involving the addition of an acetyl group to the ϵ-amino group of histone lysine residues (16). The basic amino acid side chains of histones carry positive charges and interact electrostatically with the negatively charged DNA backbone, enabling tight wrapping into nucleosomes. Once the ϵ-amino group of lysine is acetylated, this positive charge is neutralized, weakening histone–DNA interactions and increasing chromatin accessibility. This state generally favors transcriptional activation and elongation, and is associated with a more open chromatin configuration (8, 17). A dynamic equilibrium is upheld through the interaction of “writers” — known as histone acetyltransferases (HATs), which are now more frequently referred to as lysine acetyltransferases (KATs) — and “erasers,” which are the histone deacetylases (HDACs).

KATs are primarily divided into three main families: the p300/CBP group, which includes CBP and p300; the GNAT group, comprising GCN5 and PCAF; and the MYST group, which consists of TIP60, MOZ, MORF, HBO1, and MOF (18). HDACs are classified according to their catalytic mechanism into Zn²^+^-dependent and NAD^+^-dependent enzymes. The Zn²^+^-dependent classical HDACs comprise class I (HDAC1–3 and HDAC8), class II (HDAC4–7, HDAC9 and HDAC10) and class IV (HDAC11), whereas the NAD^+^-dependent enzymes are the class III sirtuins (SIRT1–7) (19). In the context of inflammatory responses, acetylation frequently serves as a swift and reversible epigenetic mechanism that modulates the expression of genes associated with inflammation. For instance, lysine acetyltransferase 2A (KAT2A) catalyzes H3K9 acetylation and affects the NRF2-related transcriptional repression axis, thereby promoting the inducible transcription of inflammatory genes such as Il1b and Nlrp3, suggesting that inhibition of HAT activity may help restrain inflammatory macrophage activation (20).

CBP and p300 are closely associated with histone H3 lysine 27 acetylation (H3K27ac) and sustain persistent inflammatory transcriptional networks (21). The pathological importance of the acetylation–fibrosis axis in skin fibrosis is becoming more apparent. In the skin of individuals with SSc, p300 is found to be upregulated and is positively influenced by transforming growth factor-β (TGF-β) (22). It contributes to a profibrotic positive-feedback loop by increasing the acetylation and expression of genes related to fibrosis, such as collagen (23). Moreover, TGF-β/Smad-driven transcription of type I collagen also requires cooperation with coactivators such as p300/CBP (23, 24). At the “eraser” end, SIRT1 expression is downregulated in SSc. Given that SIRT1 exerts antifibrotic effects by suppressing TGF-β-related transcription, this finding indicates a close association between deacetylases and the fibrotic phenotype (25). Pharmacological experiments showed that the HDAC inhibitor trichostatin A (TSA) blocks TGF-β-induced collagen production in dermal fibroblasts, thereby reinforcing the concept of the “writer–reader–eraser” machinery as a viable therapeutic target (26). In addition, bromodomain and extra-terminal domain proteins (BET) recognize acetylated lysine residues, and BET inhibition exhibits anti-skin-fibrotic effects in both SSc animal models and patient-derived fibroblasts, indicating that the “reader” arm of acetylation signaling also shapes the strength of fibrotic transcriptional programs (27).

### 2.2 Histone methylation

Methylation of histones predominantly takes place on lysine (K) and arginine (R) residues located in the N-terminal tails of the histones, existing as mono-, di-, or trimethylation forms. Additionally, arginine residues can experience either symmetric or asymmetric dimethylation. The biological implications of this modification are largely influenced by the particular residue affected and the extent of methylation (28, 29). In contrast to acetylation, methylation does not modify the charge of the residue being altered directly. Rather, it is recognized by reader proteins, including those with chromodomain or Tudor domain structures, which attract complexes to reorganize the chromatin architecture. Typically, H3K4me3, H3K36me3, and H3K79me3 correlate with transcriptional activation, while H3K9me3 and histone H3 lysine 27 trimethylation (H3K27me3) are more frequently associated with gene silencing and the maintenance of heterochromatin (8).

During inflammatory and immune responses, dynamic changes in these epigenetic marks can profoundly influence cellular phenotypes. Inflammatory stimuli induce NF-κB-dependent expression of the H3K27 demethylase Jumonji domain-containing protein D3 (JMJD3), thereby reducing local H3K27me3 levels and relieving Polycomb-mediated repression to enhance transcription of pro-inflammatory genes (30). In contrast, the enzyme Setdb1, a methyltransferase for H3K9, upholds the baseline levels of H3K9 methylation and restricts the recruitment of NF-κB to the IL6 promoter that is triggered by Toll-like receptor 4 (TLR4), thus inhibiting the expression of inflammatory factors, including IL-6 and others (31). During adaptive immune responses, the genome-wide distribution of H3K4me3 and H3K27me3 is markedly remodeled as CD4^+^ T cells differentiate into Th1, Th2, Th17 and iTreg subsets. Lineage-defining genes such as IFNG, IL4 and IL17, together with loci encoding key transcription factors, exhibit distinct methylation patterns, indicating that histone methylation contributes to both cell fate determination and the regulation of cellular plasticity (32). In systemic sclerosis, the long non-coding RNA HOTAIR can influence the Notch pathway through polycomb repressive complex 2 (PRC2)-mediated H3K27 methylation and stably upregulate GLI2 expression. Upregulation of GLI2 induces the expression of fibrotic markers such as collagen and alpha-smooth muscle actin (α-SMA). Moreover, inhibition of either enhancer of zeste homolog 2 (EZH2) or GLI2 attenuates the profibrotic phenotype of fibroblasts, suggesting that this pathway may contribute to the epigenetic maintenance of the fibrotic state (33).

### 2.3 Histone lactylation

Histone lactylation is a newly identified post-translational modification that occurs on lysine residues of histones. It provides a direct route by which cellular metabolic status, particularly lactate generated through glycolysis, is “written” into chromatin-level transcriptional regulation. Histone lysine lactylation (Kla) was first reported in 2019, with at least 28 lactylation sites identified on core histones in mammalian cells. Under conditions that enhance glycolysis, such as hypoxia or bacterial stimulation, elevated intracellular lactate promotes histone lactylation deposition and is associated with transcriptional activation (10, 34).

Histone lactylation exists in at least three structurally related forms: lysine L-lactylation (Kl-la), lysine D-lactylation (Kd-la), and N-ϵ-(carboxyethyl)lysine (Kce) (35). Recent research employing high-performance liquid chromatography along with specific antibodies has revealed that, within the histone lactylation species influenced by glycolysis, Kl-la emerges as the most prevalent variant. Therefore, an increase in observed Kla does not necessarily indicate that L-lactate directly drives epigenetic processes within the nucleus; distinguishing among isomeric sources is essential for mechanistic interpretation (36). At the enzymatic level, Kla is a reversible modification with both writer and eraser activities, although the donor substrate and catalytic route are not unique. Historically, lactyl-CoA was regarded as the primary donor, while p300/CBP and associated KATs were suggested as possible writers. However, more recent research has indicated that ACSS2 or nuclear GTPSCS can enhance the production of lactyl-CoA and facilitate histone lactylation (37, 38), whereas AARS1 can mediate lysine lactylation through a lactyl-AMP-dependent pathway, indicating that the conversion from lactate to lactylation does not rely strictly on a single lactyl-CoA route (39). On the erasure side, HDAC1–3 and SIRT2 have been shown to possess delactylase activity and can dynamically remove Kla (40, 41). In addition, S-D-lactoylglutathione (SLG) can generate Kd-la through non-enzymatic S-to-N acyl transfer, and accumulation of SLG resulting from GLO2 deficiency can alter histone lactylation. Thus, the chemical origin of Kla must be validated independently, rather than inferred solely from elevated lactate levels (42, 43).

Kla is widely regarded as an important mark of metabolic–epigenetic coupling. In macrophages, for example, lactate accumulation during the late phase of M1 polarization can act as an endogenous “lactate clock”, promoting the late induction of a subset of homeostasis-associated genes through histone lactylation and thereby contributing to inflammation resolution and tissue remodeling (10, 44). In fibrosis research, the lactate–Kla axis has been invoked to explain the activation of profibrotic transcriptional programs. For instance, histone H3 lysine 18 lactylation (H3K18la) can promote SOX9 transcription and accelerate the progression of liver fibrosis, whereas the H3K14la–KLF5 axis can drive epithelial–mesenchymal transition in renal tubular cells and aggravate renal fibrosis (45, 46). These findings provide a useful mechanistic framework for skin fibrotic diseases: in situations where local hypoxia, inflammation, and metabolic reprogramming result in the build-up of lactate, histone lactylation could function as a crucial epigenetic point that connects metabolic disturbances to the expression of profibrotic genes.

### 2.4 Other histone modifications

**Figure 1:**
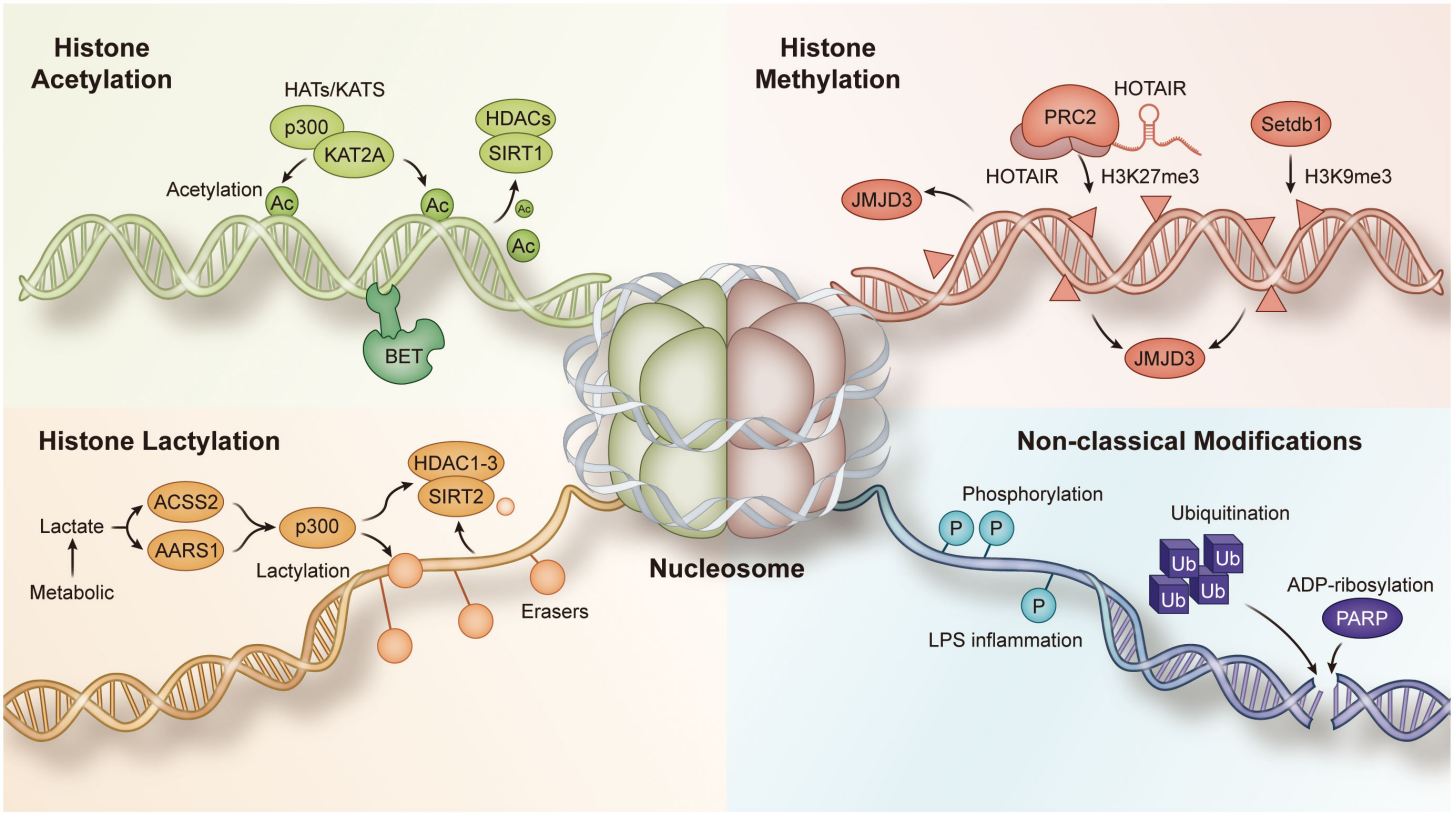
Major histone modifications and their regulatory functions.

Beyond these major histone modifications, histones may also experience various “non-canonical” modifications such as phosphorylation, ubiquitination/SUMOylation, biotinylation, and ADP-ribosylation. These modifications are often enriched on the N-terminal tails of histones and, through crosstalk with one another, collectively reshape chromatin state. Serine/threonine phosphorylation can rapidly weaken histone–DNA interactions and often acts in concert with neighboring lysine acetylation to regulate transcription, mitosis, DNA repair and apoptosis (47, 48). This mechanism is particularly prominent in inflammatory settings. For example, in mouse macrophages stimulated with bacterial lipopolysaccharide (LPS), H3S28 phosphorylation is one of the major stimulus-dependent histone marks and is enriched at inducible gene loci, where it mediates inflammatory transcriptional responses (49). Ubiquitination and SUMOylation involve covalent attachment to the ϵ-amino group of lysine residues and participate in the repair of DNA double-strand breaks (50, 51). Histone biotinylation is relatively rare and also occurs mainly on lysine residues; it may be associated with transcriptional repression (52). In addition, metabolism-related modifications such as glycosylation are attracting increasing attention, although their roles in fibrosis remain to be clarified. ADP-ribosylation, catalyzed by PARP and other ADP-ribosyltransferases using NAD^+^ as a substrate, can increase local chromatin accessibility during DNA damage and enhance the openness of promoter regions for inflammatory genes in immune cells (53–55). Overall, although direct studies of these non-canonical modifications in skin fibrosis are still limited, the chronic inflammation and DNA damage response that accompany fibrotic processes suggest that they may cooperate with canonical modifications to form a more complete epigenetic regulatory network, and therefore merit brief discussion in this review. **Figure 1** provides an overview of the major forms of histone modification and their roles in regulating chromatin structure and gene transcription.

## 3 Histone modifications in skin fibrotic diseases

### 3.1 HS and keloids

HS and keloids are both pathological scars, representing fibrotic diseases caused by abnormal wound repair after skin injury. They are characterized by persistent chronic inflammatory stimulation, fibroblast dysfunction and excessive collagen deposition (56). Clinically, both lesions arise predominantly in high-tension regions, including the anterior chest, shoulders, back and joints, where repetitive movement and mechanical stress may promote their initiation and progression (57). Pathological scars have long troubled patients, especially keloids, which are often accompanied by pruritus, pain, cosmetic deformity and restricted movement, thereby affecting patients’ confidence, self-esteem and overall quality of life. Existing treatment options, including surgical removal, corticosteroid injections, laser treatments, and silicone dressings, do not adhere to a consistent standard; their effectiveness varies, and the rates of recurrence continue to be significant. Consequently, gaining a more comprehensive understanding of their pathogenesis is crucial for pinpointing possible therapeutic targets and biomarkers.

Within this context, histone modifications can be understood as an epigenetic interface through which chronic inflammation, mechanical tension, and metabolic rewiring are converted into persistent fibroblast activation and scar-promoting transcriptional programs.

The mechanism of pathological scarring is not simply the dysregulation of a single pathway, but rather a complex, self-amplifying molecular network. As a key canonical pathway in its pathogenesis, the TGF-β/Smad signaling pathway exerts its effects primarily by regulating fibroblast proliferation, apoptosis, extracellular matrix deposition and cytokine secretion (58, 59). Moreover, signaling axes such as Yes-associated protein 1 (YAP)/transcriptional coactivator with PDZ-binding motif (TAZ) and Wnt/β-catenin are also involved in disease progression (60, 61). With advances in epigenetics, mechanistic studies in this field have become increasingly refined, providing new directions for targeted therapy. (**Table 1** Recent advances in the study of histone modifications in pathological scarring). Overall, evidence is most extensive for histone acetylation, whereas methylation remains underexplored and lactylation is supported by fewer but increasingly mechanistic studies.

**Table 1:** Recent advances in the study of histone modifications in pathological scarring.

Modification	Site	Results	Ref.
Histone acetylation	/	In a rabbit ear model, Trichostatin A attenuated hypertrophic scar formation and reduced the expression of collagen I and fibronectin	(73)
	/	Resveratrol upregulates SIRT1 expression, thereby suppressing the expression of α-SMA, COL1 and COL3 in hypertrophic scar fibroblasts	(68)
	/	Treatment of scar fibroblasts with Trichostatin A increased histone H3 acetylation and CAV1 expression, while reducing RUNX2 and fibronectin expression, and was associated with reduced cellular stiffness and migratory capacity	(74)
	/	CUDC-907 exerts anti-keloid effects through dual inhibition of HDAC activity and AKT–mTOR signaling, accompanied by increased histone H3 acetylation	(63)
	/	HDAC5 promotes pathological scar formation by enhancing TGF-β1-induced Smad2/3 phosphorylation and suppressing Smad7 expression	(66)
	H3K27ac/H3K9ac	APN-mediated upregulation of SIRT1 suppresses acetylation of C/EBPβ at K39 and of H3K27 and H3K9 in fibroblasts, thereby inhibiting YAP transcription	(69)
Histone methylation	H3K27me3	LSP1P5 exerts profibrotic effects by interacting with SUZ12 to recruit PRC2 to the CEBPA promoter, thereby enhancing H3K27me3 deposition, repressing CEBPA expression and attenuating its antifibrotic activity	(76)
Histone lactylation	H3K18la	Analysis of HS tissues and HS-derived fibroblasts showed elevated Pan-Kla and H3K18la levels relative to normal controls	(14)
	H3K18la	H3K18la is elevated in keloid fibroblasts and promotes collagen expression. This study further identified a lactate–H3K18la–LTBP3–TGF-β1 positive-feedback loop that sustains fibroblast collagen production, proliferation and migration	(79)
	H3K23la	Macrophage-derived lactate enters fibroblasts via MCT1 and induces HEY2 and COL11A1 expression; HEY2 activates YAP1/Smad2 signaling, whereas COL11A1 stabilizes MCT1	(15)

AKT, protein kinase B; mTOR, mechanistic target of rapamycin; MCT1, monocarboxylate transporter 1; H3K23la, histone H3 lysine 23 lactylation.

#### 3.1.1 Abnormal histone acetylation and targeted interventions

HDACs have also been implicated in pathological scarring. HDAC2 expression is increased in human keloid tissue and in scar tissue from the corresponding mouse model. In normal human dermal fibroblasts, TGF-β1 increased HDAC2 expression in a concentration-dependent manner, suggesting that HDAC2 may contribute to fibroblast activation (62). Another study showed that HDAC2, phosphorylated AKT and mTOR were increased in keloid tissue compared with adjacent non-lesional tissue. The dual PI3K/AKT–HDAC inhibitor CUDC-907 suppressed the proliferation, migration, invasion and extracellular matrix production of keloid fibroblasts. It also increased histone H3 acetylation and reduced Smad2/3 and ERK phosphorylation, supporting its potential therapeutic activity in keloids (63). Likewise, the dual small-molecule inhibitor corin, which concurrently inhibits LSD1 and HDAC1/2 within the CoREST, effectively curtails the proliferation and invasion of keloid fibroblasts (64, 65).

Gao et al. discovered that HDAC5 expression was increased in both a mouse HS model and human HS tissue (66). *In vivo* studies showed that HDAC5 knockdown reduced scar formation, whereas *in vitro* experiments demonstrated that HDAC5 inhibition suppressed fibroblast activation, markedly reduced TGF-β1-induced Smad2/3 phosphorylation and increased Smad7 expression. Conversely, Smad7 downregulation abrogated the antifibrotic effects associated with HDAC5 deficiency. Luciferase reporter and ChIP-qPCR assays further revealed that HDAC5 interacts with myocyte enhancer factor 2A (MEF2A) and restrains its binding to the Smad7 promoter, thereby suppressing Smad7 transcription (66). HDAC6 also merits consideration. Tubacin inhibited HDAC6-mediated deacetylation of α-tubulin and Hsp90, thereby attenuating TGF-β1-induced proliferation, myofibroblast differentiation, migration and extracellular matrix deposition in human dermal fibroblasts, thus providing a rationale for antifibrotic therapeutic strategies (67). These studies consistently implicate HDAC activity in pathological scarring, but the evidence is not equally strong across isoforms. HDAC5 is supported by *in vivo* knockdown and a defined MEF2A–Smad7 mechanism, whereas HDAC2 and HDAC6 are supported mainly by inhibitor-based and fibroblast experiments. The effects of broad HDAC inhibition should therefore not be equated with isoform-specific causality.

Previous research has indicated that SIRT1 expression is suppressed in HS tissues. Downregulation of SIRT1 increases the expression of α-SMA, collagen type I (Col1) and collagen type III (Col3) in fibroblasts derived from HS (68). By contrast, overexpression of SIRT1 not only suppresses the expression of collagen and α-SMA, but also blocks TGF-β1-induced activation of normal fibroblasts. Moreover, in wound-healing models, SIRT1 deficiency results in denser and more disorganized collagen fibers, whereas resveratrol treatment leads to finer and more orderly collagen architecture (68). Another investigation similarly verified that adiponectin (APN), a protective adipokine, was significantly diminished in both HS tissue and HS fibroblasts. Experiments revealed that APN markedly enhanced SIRT1 expression and inhibited the fibrotic characteristics and proliferation of HS fibroblasts, further reinforcing the idea that SIRT1 suppresses YAP transcriptional activation in HS fibroblasts by inhibiting acetylation at specific sites on C/EBPβ (K39) and histone H3 (K9 and K27) (69).

TSA, an inhibitor of natural histone deacetylase, has demonstrated the ability to hinder fibrogenic signaling pathways and diminish excessive deposition of extracellular matrix (70). Research by Rombouts et al. revealed that TSA reduced collagen synthesis and gene expression associated with fibrosis in rat fibroblasts, furthermore inhibiting fibrosis induced by TGF-β1 in skin fibroblasts (71). Following this, Diao et al. illustrated that TSA interrupted TGF-β1-driven collagen synthesis in keloid fibroblasts and prompted apoptosis (72). In their experiments, a 0.02% TSA solution was injected into a rabbit HS model, showing that by the 23rd day post-operation, the scars appeared more normalized, displaying reduced synthesis of Col1 and fibronectin; by the 45th day post-operation, the scar elevation index was considerably lower in comparison to the control HS group, highlighting TSA’s antifibrotic properties in relation to HS formation (73). To further explore the underlying mechanisms, Chao-Kai Hsu et al. treated keloid fibroblasts with TSA and observed increased histone H3 acetylation and CAV1 expression, together with reduced RUNX2 and fibronectin expression, as well as diminished cellular stiffness and migratory capacity (74). Jian et al. further showed that TSA inhibited keloid fibroblast proliferation in both a time-dependent and dose-dependent manner, while also altering the expression profiles of multiple miRNAs, including miR-30a-5p. Collectively, TSA shows reproducible antifibrotic effects across fibroblast and rabbit scar models. However, as a broad HDAC inhibitor that also induces apoptosis, it does not identify the responsible HDAC isoform or exclude nonspecific cytotoxic effects. The associated miRNA changes therefore indicate epigenetic crosstalk but do not independently establish a histone-dependent mechanism (75).

#### 3.1.2 Histone methylation-related profibrotic mechanisms

Shuchen Gu et al. identified a human-specific long non-coding RNA, lymphocyte-specific protein 1 pseudogene 5 (LSP1P5). In keloid tissue, LSP1P5 contributes to maintenance of the fibrotic state and to the expression of extracellular matrix components, including COL1, COL3 and fibronectin 1 (FN1). Mechanistically, LSP1P5 interacts with SUZ12 and recruits PRC2 to the CEBPA promoter, thereby promoting H3K27me3 deposition, altering chromatin accessibility and ultimately silencing CEBPA (76). Collectively, this study defines a distinct epigenetic regulatory axis that is crucial for ECM deposition and helps to fill an important gap in our understanding of the interplay among lncRNA-mediated regulation, histone modifications and ECM remodeling in keloid pathogenesis. Nevertheless, this evidence is currently dominated by a single mechanistic axis. Compared with acetylation, the general contribution of histone methylation to HS and keloid fibrosis therefore remains less firmly established.

#### 3.1.3 Histone lactylation-related profibrotic mechanisms

Meng et al. reported that glycolytic activity was markedly increased in HS and keloids, and that this increase was positively correlated with fibroblast activation. Moreover, inhibition of glycolysis attenuated fibroblast activity (77). Lactate, a glycolytic metabolite, has been established as an important regulator of epigenetic reprogramming (10). More recently, growing evidence has linked histone lactylation to fibrosis, highlighting its potential role in coupling metabolic reprogramming to profibrotic gene expression (78). Liu et al. detected increased pan-Kla and H3K18la levels in HS tissues and HS-derived fibroblasts compared with normal controls. LC3B expression was reduced, whereas p62 expression was increased, suggesting that the fibrotic phenotype may be associated with impaired autophagy. Knockdown of lactate dehydrogenase A (LDHA) improved the fibrotic phenotype, upregulated phosphatase and tensin homolog (PTEN), and reduced the phosphorylation levels of PI3K, AKT and mTOR. In addition, ChIP-qPCR and RNA immunoprecipitation (RIP) further showed that LDHA knockdown reduced H3K18la enrichment at the SLUG promoter, whereas RNA immunoprecipitation suggested that SLUG interacts with PTEN mRNA. These findings support a model in which H3K18la enhances SLUG transcription, suppresses PTEN and activates PI3K–AKT–mTOR signaling. This pathway may impair autophagy and promote fibroblast survival and collagen deposition (14). Employing cleavage under targets and tagmentation (CUT&Tag) along with RNA-seq methodologies, Gu et al. further demonstrated that in keloid fibroblasts, H3K18la was notably enriched at the promoter region of LTBP3, thereby facilitating its transcription. They subsequently confirmed that the elevation of LTBP3 levels led to an increase in TGF-β1 secretion, whereas lactate also contributed to the enhancement of TGF-β1 secretion. The silencing of LTBP3 resulted in a reduction of TGF-β1 secretion, and the introduction of recombinant TGF-β1 (rTGF-β1) counteracted the suppressive effects of si-LTBP3 on collagen production and cell proliferation. Ultimately, they illustrated that TGF-β1 additionally promoted higher levels of lactate and H3K18la, establishing a positive feedback mechanism involving lactate, H3K18la, LTBP3, and TGF-β1 (79).

**Figure 2:**
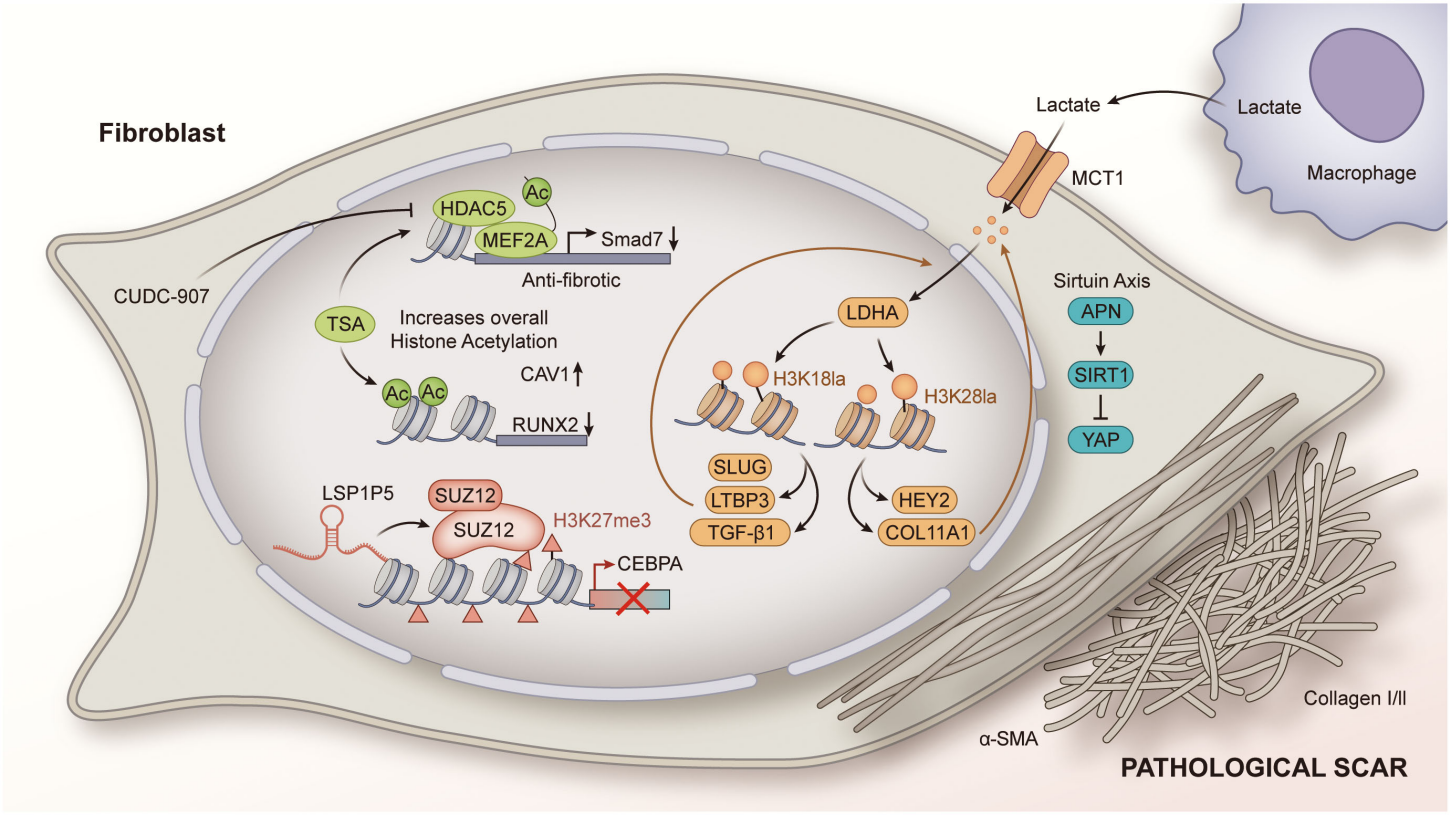
Histone modification networks in pathological scar fibroblasts.

An apparent inconsistency arises from Yuan et al., who found that histone H3 lysine 23 lactylation (H3K23la), rather than H3K18la, H3K9la, H3K14la or H3K27la, was selectively increased in HS tissue, accompanied by elevated monocarboxylate transporter 1 (MCT1) expression and higher lactate levels. Metabolic analyses indicated a glycolytic shift in HS-derived macrophages, as reflected by increased ECAR and a higher glycoATP/mitoATP ratio, and key glycolytic enzymes such as HK2 and LDHA were upregulated in CD68^+^ macrophages. Depletion of macrophages *in vivo* using clodronate liposomes reduced lactate levels in scar tissue, confirming macrophages as a major source of lactate. Moreover, treatment of conditioned-medium-induced fibroblasts with the MCT1 inhibitor AZD3965 reversed the fibrotic phenotype, and reducing upstream lactate production also rescued this phenotype. The results also indicate that lactate derived from macrophages is taken up by fibroblasts through MCT1, leading to changes in their phenotype. Analyses using CUT&Tag and ChIP-qPCR revealed an enrichment of H3K23la at the promoters of both HEY2 and COL11A1. HEY2 promotes YAP1/Smad2 signaling, which increases the profibrotic transcriptional program, while COL11A1 supports MCT1 stabilization and enhances the transport of lactate, thus creating a self-sustaining feedback loop involving lactate, H3K23la, COL11A1, and MCT1 (15). This result does not necessarily invalidate the H3K18la studies. The H3K23la study emphasizes macrophage-derived lactate and intercellular metabolic coupling, whereas the H3K18la studies focus mainly on scar fibroblasts. Differences in cell composition, scar type and detection methods may therefore explain the distinct lysine residues identified, although direct comparison in matched samples is still required. Together, these findings support convergence of histone acetylation and lactylation on persistent profibrotic signaling, whereas evidence for methylation remains comparatively limited. Acetylation currently has the broadest experimental support, while lactylation provides a plausible metabolic–epigenetic mechanism but has fewer independent studies. Because nearly all interventions remain preclinical, their efficacy and selectivity in established human scars are unresolved. **Figure 2** summarizes the histone modification networks involved in pathological scar fibroblasts, highlighting how acetylation, methylation and lactylation contribute to fibroblast activation and profibrotic signaling.

### 3.2 Systemic sclerosis

Systemic sclerosis, a type of systemic autoimmune rheumatic disorder, is primarily marked by thickening and hardening of the skin, with its three principal characteristics being inflammation, vasculopathy, and fibrosis (80). Beyond affecting the skin, this condition can also have an impact on organs such as the lungs, kidneys, the cardiovascular system and the gastrointestinal tract. As in many autoimmune disorders, SSc is predominantly observed in women; nevertheless, male patients frequently experience more severe manifestations, including a greater tendency for diffuse cutaneous involvement and pulmonary arterial hypertension, and consequently a poorer prognosis (81). According to the extent of skin involvement, SSc is classified into four subtypes: diffuse cutaneous SSc, limited cutaneous SSc, sine scleroderma SSc and overlap syndrome (3). The underlying mechanisms of SSc are intricate. Despite notable advancements in understanding these processes over the last few decades, there remains an absence of targeted therapies capable of changing its progression towards fibrosis. This lack of treatment options may be attributed to the interplay of genetic predisposition, environmental influences, and epigenetic modifications (12). In this autoimmune and vasculopathic setting, histone modifications appear to integrate immune-cell abnormalities with fibroblast-intrinsic profibrotic programs.

Wang et al. reported global histone H4 hyperacetylation and H3K9 hypomethylation in peripheral B cells from patients with SSc. These changes were accompanied by reduced expression of HDAC2, HDAC7 and SUV39H2, together with increased expression of JHDM2A (82). Additionally, another investigation indicated that in monocytes affected by SSc, 1,046 genomic locations presented abnormal modifications of H3K4me3, and 534 exhibited changes in H3K27ac. Furthermore, the expression of 381 genes was positively correlated with the levels of chromatin modifications in regions surrounding their transcription start sites. Genes associated with these altered histone marks were predominantly enriched in pathways related to immune responses and interferon. Moreover, global H3K27me3 levels were markedly reduced in CD4+ T cells from patients with SSc (83). Collectively, these findings suggest that histone modifications contribute to the pathogenesis of SSc and may provide a rationale for the development of novel therapeutic strategies (84). (**Table 2** Recent advances in the study of histone modifications in systemic sclerosis-associated fibrosis). These studies consistently demonstrate chromatin dysregulation in SSc immune cells, particularly at immune-response and interferon-related loci. However, they are mainly associative and do not establish that these changes directly drive dermal fibrosis; their cell-type specificity also limits interpretation of global histone-mark changes.

**Table 2:** Recent advances in the study of histone modifications in systemic sclerosis-associated fibrosis.

Modification	Site	Results	Ref.
Histone acetylation	/	In fibrotic models, Trichostatin A attenuates fibrosis by preventing WIF-1 loss, β-catenin induction and collagen accumulation	(95)
	/	SIRT1 has antifibrotic properties, and its reduced expression in SSc tissues may contribute to fibrotic progression	(25)
	/	Trichostatin A significantly reduced miR-125b expression in dermal fibroblasts, and miR-125b knockdown similarly inhibited cell proliferation and α-smooth muscle actin (α-SMA) expression	(96)
	H4K16ac	TGF-β induces autophagy in fibrotic disease through downregulation of the H4K16 histone acetyltransferase MYST1, whereas restoration of H4K16ac suppresses autophagy and restrains the profibrotic effects of TGF-β	(97)
	/	Silica promotes HDAC4 expression in fibroblasts, thereby enhancing Smad2/3 phosphorylation and upregulating COL1, α-SMA and CTGF expression, ultimately exacerbating skin fibrosis in SSc	(90)
Histone methylation	H3K27me3	Inhibition of H3K27me3 induced fra-2 expression in both *in vitro* and *in vivo* settings, and fra-2 deletion completely abrogated the profibrotic effects of DZNep	(99)
	/	Fra-2 upregulation was detected in skin biopsy specimens from both bleomycin-treated mice and patients with SSc. Moreover, co-treatment of monocytes with DZNep and TLR-8 stimulation induced marked α-SMA expression in dermal fibroblasts.	(100)
	H3K27me3	JMJD3 regulates fibroblast activation by modulating H3K27me3 levels at the fra-2 promoter, and its targeted inhibition exerts antifibrotic effects in murine models	(101)
	H3K27me3	EZH2 and H3K27me3 are both upregulated in dermal fibroblasts and endothelial cells from patients with SSc. DZNep-mediated inhibition of EZH2 reduces profibrotic gene expression and suppresses the migratory capacity of SSc fibroblasts	(98)
	H3K27me3	HOTAIR mediates EZH2-dependent H3K27me3 deposition at specific target genes in fibroblasts, thereby increasing collagen and α-SMA expression and further activating NOTCH signaling, which together sustain the profibrotic phenotype	(33)

CTGF, connective tissue growth factor.

#### 3.2.1 Histone acetylation and targeted interventions

Wang and colleagues initially indicated that inhibitors of epigenetic processes might return collagen expression in SSc fibroblasts to typical levels. This effect appears to be associated with the epigenetic silencing of the collagen-suppressive gene FLI1, primarily via DNA methylation and histone deacetylation at its locus (85). Noda et al. additionally verified that, beyond the alterations in methylation of the promoters of the epigenetic repressors FLI1 and KLF5, fibroblasts from SSc showed significantly reduced levels of histone acetylation at the KLF5 promoter compared to normal fibroblasts (86). A study on chromatin accessibility and transcriptomic profiling discovered an enhancer for TGF-β2 in SSc that is triggered by epigenetic memory. The TGF-β2 signaling pathway that follows preserves a profibrotic condition in fibroblasts *in vitro*. This enhancer exhibits epigenetic characteristics of active enhancers, such as elevated H3K27ac and strengthened binding of p300 (87). As a competitive inhibitor of BRD4, JQ1 disrupts its interaction with acetylated lysine residues and durably suppresses TGF-β2 enhancer activity, attenuates profibrotic gene expression and reverses dermal fibrosis in patient-derived skin explants (87). As early as 2005, Bhattacharyya and colleagues reported that p300 expression in fibroblasts from patients with SSc was twofold to threefold higher than that in normal controls (88). This observation was subsequently confirmed by Ghosh and colleagues, who showed increased p300 expression in skin biopsies from patients with SSc and further demonstrated that TGF-β induces p300 in an Smad-independent manner through early growth response 1 (EGR1) (23). EGR1 is a key mediator of TGF-β signaling and is also upregulated in SSc skin biopsies (89). In addition, TGF-β promotes the recruitment of p300 to the COL1A2 promoter, thereby enhancing histone H4 acetylation, which suggests that p300-driven histone acetylation contributes to fibrosis (23). More recently, Tang and colleagues discovered that low-dose silica aggravated skin fibrosis associated with SSc by increasing levels of HDAC4 and activating the Smad2/3 signaling pathway, implying that HDAC4 could serve as a promising therapeutic target (90).

SIRT1 is generally downregulated in SSc skin tissue, but its regulatory role in fibrosis remains controversial. Wei et al. identified SIRT1 as a negative regulator of fibrotic transcription. Activation or overexpression of SIRT1 reduced p300 abundance, weakened the interaction between p300 and Smad2/3, and decreased p300 recruitment and histone H4 acetylation at the COL1A2 promoter, thereby suppressing collagen transcription and fibrotic responses (25). By contrast, Zerr et al. reported that SIRT1 activation enhanced TGF-β-induced Smad reporter activity and collagen release, whereas fibroblast-specific inactivation of SIRT1 reduced skin thickening, myofibroblast accumulation, hydroxyproline deposition and Smad2/3 phosphorylation in bleomycin-induced and constitutively active TGF-β receptor I (TBRIact)-driven models of skin fibrosis (91). The reasons for these apparently divergent findings remain unclear. Several non-mutually exclusive factors may contribute. SIRT1 regulates multiple histone and non-histone substrates, and its net effect may depend on whether p300-dependent chromatin regulation or canonical Smad signaling predominates in a given experimental setting. Differences between pharmacological activation and genetic inactivation, as well as variations in fibroblast state, TGF-β signaling intensity, experimental model and disease stage, may also influence the observed outcome. These possibilities have not yet been directly compared and therefore require further experimental validation. Overall, SIRT1 should be regarded as a context-dependent regulator rather than as a uniformly profibrotic or antifibrotic factor. Notably, fibroblast-specific genetic inactivation provides stronger causal evidence than pharmacological activation or overexpression, but the divergent models still preclude defining SIRT1 as a uniformly safe therapeutic target. In addition to SIRT1, SIRT3 is markedly downregulated in SSc. Akamata et al. showed that hexafluoro, a novel fluorinated synthetic honokiol analog with SIRT3 agonist activity, restored SIRT3 expression, reduced mitochondrial and cytosolic reactive oxygen species (ROS) accumulation in TGF-β-treated fibroblasts, and ameliorated bleomycin-induced skin fibrosis. These findings support pharmacological enhancement of SIRT3 as a potential antifibrotic strategy (92).

Early studies showed that TSA reduced collagen I and fibronectin levels in TGF-β-stimulated skin fibroblasts from both healthy controls and patients with SSc (85). TSA also reduced overall collagen expression in SSc skin fibroblasts to baseline levels (93). Hemmatazad et al. found that TSA markedly inhibited HDAC7 expression, and that silencing HDAC7 more specifically reduced the production of type I and type III collagen while avoiding the induction of the expression of ICAM-1 and connective tissue growth factor (CTGF) (94). Moreover, TSA inhibits a well-known SSc pathway involving Wnt/β-catenin. Svegliati et al. found that the expression of the gene encoding Wnt inhibitory factor 1 (WIF-1) was reduced in the skin tissues of patients with SSc, thereby leading to activation of the downstream WNT effector β-catenin and collagen production, and TSA was able to block this process (95). In addition, Kozlova et al. found that miR-125b expression was downregulated in SSc tissues relative to controls, whereas TSA significantly reduced miR-125b expression in fibroblasts; further evidence showed that knockdown of miR-125b inhibited cell proliferation and the expression of α-SMA (96). Zehender et al. further reported that autophagy is activated in both SSc and experimental fibrosis. TGF-β induced autophagy through downregulation of the histone acetyltransferase MYST1, thereby promoting fibroblast-to-myofibroblast transition, collagen secretion and tissue fibrosis, whereas TSA inhibited this process (97). Collectively, broad HDAC inhibition produces largely consistent antifibrotic effects in SSc fibroblasts. However, the proposed downstream mechanisms are heterogeneous, and the miR-125b findings are counterintuitive because TSA further reduced a miRNA that was already decreased in SSc. These observations favor isoform-selective and cell-specific studies over further reliance on broad HDAC inhibitors.

#### 3.2.2 Histone methylation-related profibrotic mechanisms

**Figure 3:**
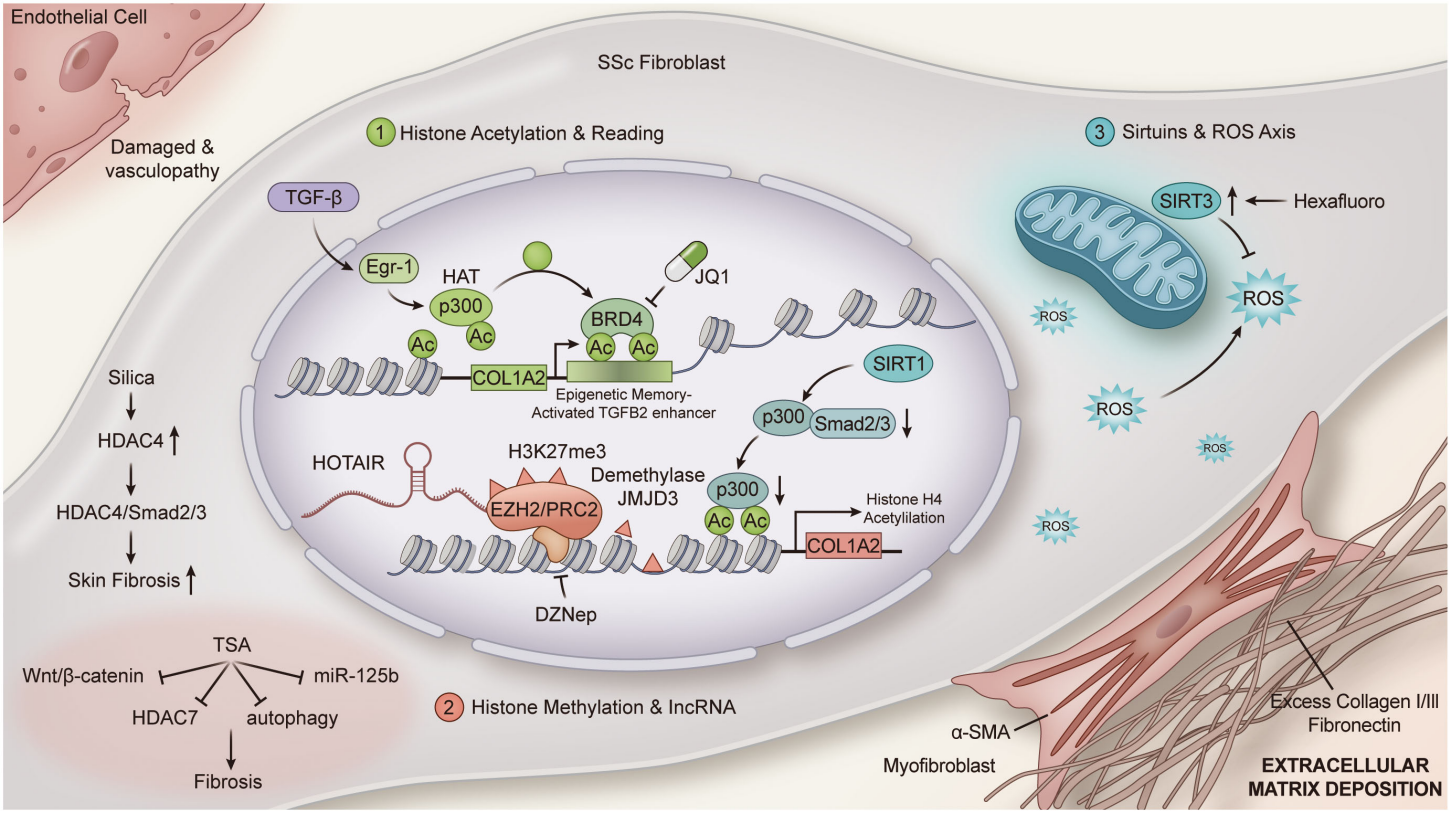
Epigenetic regulation of fibrosis in systemic sclerosis.

EZH2, as the catalytic subunit of PRC2, catalyzes H3K27me3 to mediate transcriptional repression. Research by Tsou et al. indicated that there was an upregulation of EZH2 in dermal fibroblasts and endothelial cells from patients with SSc compared to control subjects, with a notable increase in H3K27me3 levels observed in fibroblasts as well. The study found that in SSc fibroblasts, the application of the EZH2 inhibitor DZNep diminished the expression of profibrotic genes and hindered cell migration in a dose-dependent manner while also reducing H3K27me3 within a bleomycin-induced model (98). Conversely, Krämer et al. discovered that DZNep aggravated bleomycin-induced fibrosis or overexpression of a constitutively active TGF-β1, noting that DZNep alone could initiate fibrosis; this profibrotic response was mediated through the induction of fra-2, as knocking down fra-2 entirely negated the profibrotic effects of DZNep (99). H3K27me3 perturbation in SSc appears to be highly context-dependent, with effects varying by cell type, disease stage and target gene network. The opposing DZNep results constitute a genuine contradiction: reduction of H3K27me3 was antifibrotic in one study but induced fra-2-dependent fibrosis in another. DZNep-based findings alone therefore cannot define EZH2 as uniformly profibrotic or protective. The JMJD3 and HOTAIR studies instead indicate that the outcome depends on the locus and transcriptional network affected. Additionally, another investigation revealed that DZNep worked in synergy with TLR8 stimulation in monocytes to significantly enhance α-SMA production in dermal fibroblasts, thereby facilitating their differentiation into myofibroblasts. Furthermore, the demethylase JMJD3 is upregulated in both SSc dermal fibroblasts and TGF-β-induced fibrotic models (100). Inhibition of JMJD3 reversed the activated fibroblast phenotype, primarily by promoting H3K27me3 accumulation at the fra-2 promoter, thereby exerting antifibrotic effects (101). Another pivotal discovery was the elevated expression of the lncRNA HOTAIR in SSc skin. HOTAIR was shown to recruit EZH2, leading to enhanced H3K27me3 at specific target genes, reduced miR-34a levels and subsequent activation of NOTCH signaling. Finally, EZH2 inhibition restored miR-34a expression and attenuated the profibrotic phenotype of SSc fibroblasts *in vitro*, particularly in HOTAIR-overexpressing fibroblasts (33). Overall, SSc has the broadest evidence base among the diseases discussed, spanning patient cells, omics analyses, skin explants and animal models. Nevertheless, the conflicting SIRT1 and H3K27me3 findings show that global activation or inhibition of an epigenetic regulator may produce context-dependent effects. Locus-specific mechanisms and genetic or rescue experiments therefore provide more robust evidence than global histone-mark changes or inhibitor responses alone. **Figure 3** illustrates the epigenetic mechanisms underlying fibrosis in systemic sclerosis, with particular emphasis on the roles of histone acetylation and H3K27 methylation in maintaining the profibrotic phenotype.

### 3.3 Lymphedema

Lymphedema is a chronic, progressive disorder that is classified as either primary or secondary on the basis of its underlying etiology. Primary lymphedema typically arises from congenital defects or dysfunction within the lymphatic system, while secondary lymphedema generally stems from compromised lymphatic drainage caused by cancer surgery, trauma, infections, or various other factors. The condition is characterized by inflammation in the subcutaneous tissue, swelling, fibrotic fat deposition, and a heightened susceptibility to infections. Chronic swelling and impaired limb function impose a substantial physical and psychological burden on affected individuals. Because lymphedema remains difficult to treat, it can substantially impair quality of life. Epidemiological studies suggest that approximately 250 million people worldwide are affected by lymphedema, with the population suffering from cancer-related lymphedema increasing in recent years due to the rising occurrence of cancers (102). Compared with pathological scars and systemic sclerosis, evidence in lymphedema remains more limited; however, available studies suggest that histone modifications may link lymphatic dysfunction, chronic inflammation, and fibroadipose remodeling.

HDAC3 is expressed in multiple tissues and in lymphatic endothelial cells within developing valves and the mesenteric and peripheral lymphatic vasculature. This enzyme plays a crucial role in the formation of lymphatic valves and the maturation process of mesenteric lymphatic valves. When these physiological processes are disrupted, lymphatic function is impaired, thereby reducing the capacity to transport lymph fluid effectively and ultimately leading to lymphedema (103). This evidence is mechanistically strong for lymphatic valve development, but it is not direct evidence that HDAC3 drives fibrosis in established lymphedema. Its relevance is primarily to lymphatic endothelial mechanotransduction and impaired drainage, which may indirectly promote fibrotic remodeling. Oscillatory shear stress (OSS) induces the expression of key valve-development genes in lymphatic endothelial cells (LECs), including GATA2, FOXC2 and ITGA9, with GATA2 acting as an upstream transcriptional regulator of this program (104–107). In response to OSS, HDAC3 recruits EP300 to an intragenic enhancer of GATA2, thereby promoting GATA2 transcription. This process is accompanied by H3K27ac enrichment in LECs, suggesting that lymphatic valve formation and lymphatic endothelial development are closely linked to histone acetylation (103).

**Figure 4:**
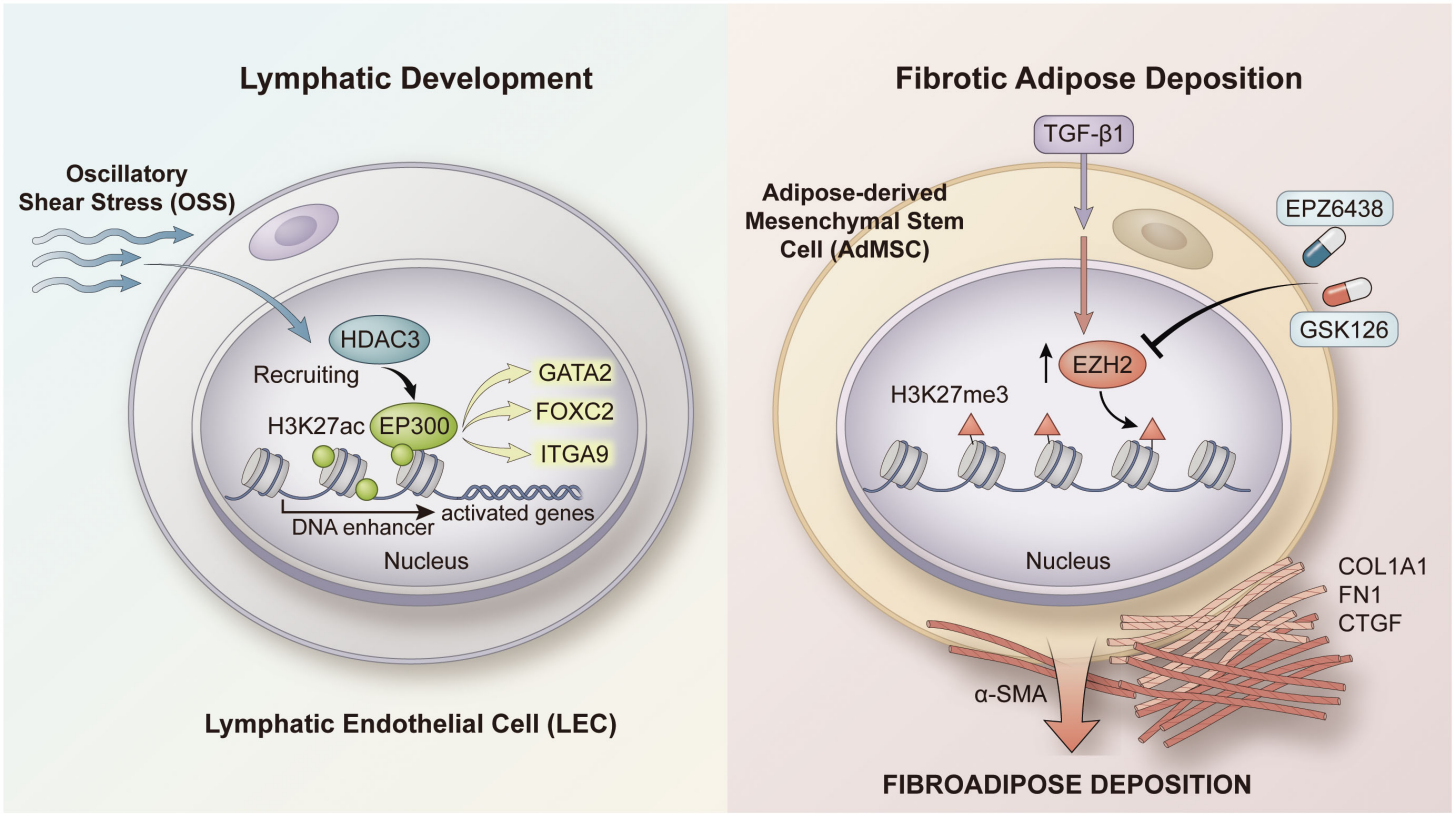
Histone modifications in lymphedema: from lymphatic development to fibroadipose deposition.

Research on lymphedema has largely focused on cutaneous fibrosis and fibroadipose deposition, with emphasis placed on improving subjective symptoms and reducing limb volume; however, the mechanism by which epigenetic reprogramming drives fibrosis has remained unresolved. In lymphedema patient skin tissue, EZH2 and H3K27me3 are significantly upregulated and are mainly localized in the nucleus. Prior research has suggested a role for EZH2 in the development of liver fibrosis, lung fibrosis, kidney fibrosis, and heart fibrosis (108–111). *In vivo* experiments showed that the EZH2 inhibitors EPZ6438 and GSK126 alleviated skin thickening and fibrotic tissue thickness, while reducing EZH2 and H3K27me3 levels. Moreover, inhibition of EZH2 attenuated TGF-β1-induced fibrotic differentiation of human adipose-derived mesenchymal stem cells (AdMSCs) *in vitro*, accompanied by reduced expression of fibrosis-associated genes, including COL1A1, FN1, CTGF and α-SMA, as well as decreased H3K27me3 levels. These findings provide functional evidence that histone methylation may contribute to fibroadipose remodeling in lymphedema (112). Research on histone modifications in lymphedema has focused primarily on methylation, while the connections between other histone modifications and lymphedema are still largely uninvestigated. Among the studies summarized here, EZH2 inhibition provides the most direct functional link between a histone modification and lymphedema-associated fibrosis because it was examined in both an animal model and human AdMSCs. However, the overall evidence remains substantially less mature than that for pathological scars or SSc and is concentrated on a single methylation axis. Current studies therefore support separate roles for HDAC3 in lymphatic valve biology and EZH2 in fibrotic differentiation, rather than a unified epigenetic mechanism of lymphedema. **Figure 4** illustrates how histone acetylation contributes to lymphatic valve development, whereas EZH2-mediated H3K27 methylation promotes fibroadipose deposition in lymphedema.

## 4 Discussion

Advances in epigenetic research have broadened the conceptual framework of skin fibrosis beyond transient inflammation and aberrant cytokine signaling to encompass persistent chromatin reprogramming. Across pathological scars, systemic sclerosis, lymphedema and other fibrotic skin disorders, altered histone acetylation, methylation and, more recently, lactylation have been linked to fibroblast activation, immune dysregulation, excessive extracellular matrix deposition and tissue remodeling. However, the available evidence does not support a universal model in which histone modifications independently initiate fibrosis. Instead, these modifications generally arise in response to inflammatory, mechanical, hypoxic or metabolic stress and subsequently reinforce profibrotic pathways, including TGF-β/Smad, YAP/TAZ and Wnt/β-catenin signaling. Histone modifications are therefore best viewed as context-dependent integrators and stabilizers of profibrotic cell states rather than as autonomous initiating lesions.

This distinction between initiation and maintenance remains central to the interpretation of the field. Most human studies are cross-sectional and examine established lesions, allowing associations between histone marks and disease severity to be identified but providing little information about temporal sequence. Observed epigenetic alterations may precede matrix accumulation, emerge during disease progression or reflect changes in the cellular composition of fibrotic tissue. Although time-course and perturbation studies indicate that chromatin remodeling can occur before overt extracellular matrix deposition, particularly after mechanical, inflammatory or metabolic stimulation, direct evidence that a specific histone modification is sufficient to initiate cutaneous fibrosis remains scarce. A more plausible model is that distinct modifications operate at different stages: some may prime cells for an exaggerated response to injury, whereas others consolidate transcriptional memory and maintain fibrosis after the initiating stimulus has diminished.

The relative importance of individual histone modifications must also be interpreted cautiously. Histone acetylation has the broadest and most consistent evidence base across cutaneous fibrotic disorders. Studies targeting HDACs, p300/CBP and BET proteins indicate that acetylation-dependent chromatin accessibility supports the expression of multiple profibrotic genes and can be pharmacologically manipulated. Nevertheless, the greater volume of evidence and therapeutic tractability of acetylation do not establish it as the biologically dominant modification. Histone methylation may confer more durable transcriptional activation or repression, but the functions of regulators such as EZH2 and JMJD3 vary according to disease, cell type, genomic locus and stage of fibrosis. In some settings, the same regulator has been associated with apparently opposing effects, emphasizing that enzyme abundance and global modification levels cannot be interpreted independently of locus-specific occupancy and cellular context.

Similarly, no fixed hierarchy among histone modifications has been established in skin fibrosis. One possible framework is that extracellular stress alters cellular metabolism and the availability of epigenetic substrates and cofactors, thereby modifying the activities of histone writers, erasers and readers. Acetylation may initially create a permissive chromatin environment that facilitates transcription-factor binding, whereas selected methylation states may subsequently stabilize longer-lasting transcriptional programs. However, this sequence is unlikely to apply uniformly across diseases or cell states. Acetylation, methylation and lactylation can influence one another and interact with DNA methylation, non-coding RNAs, nucleosome positioning and chromatin accessibility. The epigenetic regulation of fibrosis is therefore more accurately represented as an interconnected and dynamic network than as a linear cascade governed by a single dominant modification.

The extent to which histone modification profiles are disease specific also remains unresolved. Pathological scars, systemic sclerosis and lymphedema converge on TGF-β activation, myofibroblast accumulation and excessive extracellular matrix production, suggesting the existence of a shared profibrotic epigenetic core. However, these conditions differ markedly in their initiating insults, immune microenvironments, vascular abnormalities, mechanical contexts and disease trajectories. Their epigenetic landscapes are therefore likely to combine conserved profibrotic programs with disease-specific, stage-specific and cell-state-specific features. Robust comparisons are currently limited because most studies investigate a single disorder, cell population or histone modification using distinct experimental platforms and sampling strategies. Claims of disease-specific epigenetic signatures should consequently remain provisional until they are supported by standardized cross-disease and longitudinal analyses.

Histone lactylation illustrates the distinction between a compelling mechanistic framework and an established pathogenic pathway. Lactylation offers a plausible molecular link between increased glycolysis, local lactate accumulation and sustained profibrotic transcription, particularly in tissues exposed to hypoxia, chronic inflammation and altered mechanical stress. However, evidence in cutaneous fibrosis remains restricted to a limited number of models, histone sites and candidate regulatory enzymes. Moreover, interventions that reduce glycolysis, lactate production or lactate transport alter numerous metabolic and signaling pathways independently of histone lactylation. Their antifibrotic effects therefore cannot, in isolation, establish lactylation as the responsible chromatin mechanism. At present, histone lactylation should be considered a promising metabolism–chromatin interface that warrants rigorous causal testing, rather than a validated central driver of skin fibrosis.

Interpretation of the existing literature is further constrained by the experimental systems on which many mechanistic conclusions are based. *In vitro* studies frequently expose isolated fibroblasts to high concentrations of TGF-β or other single stimuli over short periods, whereas human skin fibrosis develops through prolonged interactions among stromal, immune, vascular and epidermal compartments. Animal models reproduce selected aspects of fibrogenesis but rarely capture the chronicity, tissue architecture, immune heterogeneity and spontaneous persistence of human disease. Acute wound-healing models may preferentially identify pathways involved in transient repair rather than stable fibrotic memory, whereas analyses of late-stage human biopsies are confounded by disease duration, anatomical location, treatment exposure and changes in cellular composition.

These limitations are particularly important when distinguishing association from causation. In several studies, causal mechanisms have been inferred from parallel changes in enzyme expression, global histone modification levels and fibrosis markers. Such observations remain associative unless supported by temporal ordering, genetic or pharmacological perturbation, locus-resolved chromatin analysis and, ideally, rescue experiments. Similarly, a global increase in a histone mark does not demonstrate that the modification directly regulates the profibrotic genes under investigation. Evidence should therefore be graded according to experimental strength: co-expression and bulk modification data are primarily hypothesis generating, whereas stronger causal inference requires site-specific manipulation, direct target engagement and functional reversal of the phenotype.

The therapeutic implications of histone modification research likewise require a balanced assessment. Inhibitors of HDACs, BET proteins, p300/CBP and histone methyltransferases have produced antifibrotic effects in preclinical models, but many of these agents broadly alter transcription across multiple tissues and cell populations. Systemic treatment may therefore cause hematological toxicity, hepatotoxicity, immune dysfunction, impaired tissue repair and disruption of physiological gene regulation. Experience with epigenetic inhibitors in oncology and inflammatory diseases has also shown that substantial preclinical activity does not necessarily translate into durable clinical benefit, owing to dose-limiting toxicity, incomplete target selectivity and narrow therapeutic windows. These concerns are particularly relevant to chronic fibrotic diseases outside the oncology setting, for which the acceptable threshold for systemic toxicity is considerably lower than that in advanced malignancy.

Biodistribution represents an additional translational barrier. Systemically administered agents may achieve inadequate concentrations in fibrotic skin while simultaneously perturbing epigenetic programs in the bone marrow, liver and immune system. Local injection, topical formulations, biomaterial-based delivery and cell-targeted nanoparticles could improve tissue selectivity, but each introduces additional challenges related to tissue penetration, cellular uptake, manufacturing reproducibility and long-term safety. Regulatory assessment will also need to consider the reversibility and duration of chromatin changes, potential effects on wound healing and immune surveillance, off-target transcriptional consequences and the current lack of validated pharmacodynamic biomarkers. Thus, although epigenetic therapies are mechanistically attractive, their clinical development for cutaneous fibrosis remains at an early stage.

Collectively, histone modifications should not be regarded as passive by-products of fibrosis. The available evidence supports a model in which they integrate inflammatory, metabolic and mechanical inputs and stabilize selected profibrotic transcriptional programs. Histone acetylation has the most extensive and therapeutically developed evidence base, histone methylation appears highly dependent on genomic and cellular context, and histone lactylation remains an emerging mechanism that requires direct causal validation. Establishing when, where and in which cell populations these modifications become functionally indispensable will be essential for defining their pathogenic importance and therapeutic tractability.

## 5 Future perspectives

A major priority is to distinguish epigenetic alterations that contribute to the initiation of fibrosis from those required for its persistence. Longitudinal studies should map chromatin changes before, during and after overt extracellular matrix accumulation and combine these measurements with temporally controlled genetic or pharmacological interventions. Inducible models will be particularly informative for determining whether inhibition of a specific writer, eraser or reader prevents disease onset, reverses established fibrosis or merely suppresses downstream marker expression. Rescue experiments and interventions performed after withdrawal of the initiating stimulus will be necessary to establish whether a candidate modification maintains fibrotic memory.

Resolving cellular, spatial and temporal heterogeneity will be equally important. Single-cell and spatial transcriptomic approaches should be integrated with CUT&Tag, ChIP-seq, ATAC-seq and related chromatin-profiling technologies to define histone modifications within discrete fibroblast states, immune populations, vascular cells and epidermal compartments. Standardized longitudinal and cross-disease studies could then distinguish conserved profibrotic epigenetic programs from disease-specific and stage-specific signatures. Such analyses may also identify chromatin states associated with reversible fibroblast activation, persistent fibrotic memory or treatment resistance.

Mechanistic studies must move beyond global measurements of histone marks. Future work should identify the relevant writers, erasers and readers, map their genomic targets and determine how they cooperate with disease-relevant transcription factors. For histone lactylation, isotope tracing, site-specific histone manipulation, locus-resolved chromatin profiling and metabolic rescue experiments will be required to separate direct chromatin-mediated effects from the broader biological actions of lactate. Comparable mechanistic standards should be applied to histone acetylation and methylation, particularly when the same enzyme has divergent effects across cell types or disease stages.

More physiologically representative experimental systems will also be required. Human skin organoids, ex vivo tissue cultures, multicellular co-culture systems and chronic mechanical, inflammatory or hypoxic models may better reproduce interactions among fibroblasts, immune cells, endothelial cells, keratinocytes and extracellular matrix components than conventional monocultures. Integration of these platforms with lineage tracing, spatial profiling and repeated sampling could clarify whether histone modifications merely accompany fibroblast activation or actively stabilize pathological cell states after the initiating stimulus has been removed.

Therapeutic development should increasingly move from broad epigenetic inhibition towards cell-selective, pathway-selective or locus-selective modulation. Local administration, topical delivery, injectable biomaterials and fibroblast- or immune-cell-targeted nanoparticles may reduce systemic exposure and improve the therapeutic index. However, these approaches will require systematic evaluation of tissue penetration, biodistribution, duration of target engagement and effects on normal wound repair and immune surveillance. Pharmacodynamic biomarkers will be essential both to confirm target modulation and to identify patients whose disease is driven by the relevant epigenetic program.

Finally, clinical translation will require more stringent evidentiary and regulatory standards. Preclinical studies should assess long-term toxicity, reversibility of chromatin alterations, immune and reproductive effects, carcinogenic risk and interactions with existing antifibrotic treatments. Because complete and sustained inhibition of an epigenetic regulator may disrupt physiological gene regulation, partial, transient or combination-based approaches may be more feasible than prolonged systemic blockade. Combining epigenetic interventions with anti-inflammatory, anti-TGF-β or metabolism-directed therapies could also permit lower doses and reduce pathway redundancy. Progress will therefore depend less on cataloging additional histone marks than on identifying which alterations are causal, druggable and sufficiently selective to be targeted safely in human skin fibrosis.
